# Association of Stress Renography-Derived Renal Functional Reserve With Transplant Outcomes in Recipients of Kidneys From Living Donors With a Suboptimal Glomerular Filtration Rate

**DOI:** 10.7759/cureus.115490

**Published:** 2026-08-31

**Authors:** Maneesh Kumar, Rohit Dubey, Rajkumar S Jat

**Affiliations:** 1 Urology, Dr. Rai Hospital, Sagar, IND; 2 Surgery, Government Medical College, Satna, IND; 3 Surgery, Gandhi Medical College, Bhopal, IND

**Keywords:** delayed graft function, glomerular filtration rate, graft function, kidney transplantation, living donor evaluation, living kidney donation, renal functional reserve, stress renography

## Abstract

Background: Living donor kidney transplantation remains a cornerstone of therapy for end-stage renal disease (ESRD), yet accurately characterizing the functional capacity of donors with suboptimal glomerular filtration rate (GFR) is difficult. Conventional renal function indices are static and do not capture the kidney’s adaptive capacity. Renal functional reserve (RFR), a dynamic parameter derived from stress renography, may provide additional information for donor evaluation and prediction of transplant outcomes.

Methodology: In this prospective observational study, 120 living donor-recipient pairs were evaluated. Donors with a pre-donation estimated GFR (eGFR) of 60-89 mL/min/1.73 m² underwent conventional renal assessment and stress renography to determine RFR. Recipient renal function and transplant outcomes were followed for 12 months. Multivariable linear regression, receiver operating characteristic (ROC) curve analysis, and Kaplan-Meier survival analysis were used to evaluate associations between RFR and transplant outcomes.

Results: Stress renography demonstrated an increase in GFR from 74.6 ± 8.1 to 89.8 ± 10.4 mL/min/1.73 m², yielding a mean RFR of 15.2 ± 4.3 mL/min/1.73 m². Higher donor RFR was associated with lower recipient serum creatinine during the early postoperative period and higher recipient eGFR during follow-up. In multivariable linear regression, RFR was independently associated with recipient eGFR at 12 months (standardized β = 0.487, p < 0.001; model R² = 0.45). RFR demonstrated a higher AUC for delayed graft function than baseline eGFR and serum creatinine; the difference between RFR and baseline eGFR was statistically significant by the DeLong test (p = 0.014).

Conclusions: Stress renography-derived RFR was independently associated with recipient renal function beyond conventional renal function assessment in living kidney donors with suboptimal baseline eGFR. Higher RFR was associated with better recipient eGFR at 12 months and demonstrated greater discrimination of delayed graft function than baseline eGFR. Larger multicenter studies with longer follow-up are required to validate these findings before routine clinical implementation.

## Introduction

Kidney transplantation is the treatment of choice for end-stage renal disease (ESRD), offering superior survival, better quality of life, and greater cost-effectiveness than long-term dialysis. To offset the persistent shortage of organs, living donor transplantation is widely used to broaden the donor pool and shorten waiting times [1]. As a result, transplant programs increasingly consider candidates who were previously regarded as marginal, including older donors and those with borderline kidney function [2]. This trend underscores the need for accurate donor evaluation tools that protect donor safety while securing favorable recipient outcomes [3].

Contemporary donor assessment relies on serum creatinine, estimated glomerular filtration rate (eGFR), and split renal function measured by radionuclide renography [4]. These measures, however, reflect kidney function under resting conditions and do not capture the organ’s capacity to adapt to increased demand. Donor selection becomes especially uncertain when GFR falls within the 60-89 mL/min/1.73 m² range, where the wide margin leaves the decision open to interpretation [5].

Renal functional reserve (RFR) denotes the kidney’s ability to raise its filtration rate in response to physiological or pharmacological stimuli and is regarded as a robust indicator of nephron reserve and functional capacity [6]. Stress renography assesses renal function dynamically under conditions of augmented filtration produced by physiological stimulation [7]. A low RFR may signal diminished nephron reserve despite a normal resting GFR and is encountered more frequently in older, hypertensive, or obese donors. Despite this physiological rationale, RFR is not part of routine donor evaluation, largely because of a lack of prospective data and standardized testing protocols [8].

Given the growing use of donors with suboptimal GFR and the limited ability of conventional renal function tests to predict donor adaptation and transplant outcomes, additional biomarkers are needed [9]. The present study therefore evaluates the association of stress renography-derived RFR with recipient renal and graft outcomes in living kidney donors with suboptimal baseline GFR. The primary objective was to determine whether stress renography-derived RFR was independently associated with recipient eGFR at 12 months after living donor kidney transplantation. Secondary objectives were to assess the association of donor RFR with early recipient graft function, compare the discriminatory performance of RFR with conventional donor renal function parameters for delayed graft function, and evaluate the association between RFR and graft survival during the 12-month follow-up.

## Materials and methods

Study design

This prospective observational study assessed the association of stress renography-derived RFR with early and mid-term transplant outcomes in living kidney donors with suboptimal baseline GFR and compared RFR with conventional donor renal function parameters. This study was designed and reported in accordance with the Strengthening the Reporting of Observational Studies in Epidemiology (STROBE) statement for observational studies [10].

Study setting

The study was conducted in the departments of nephrology and renal transplant surgery at Fortis Hospital, Mohali, a tertiary care hospital with an active living-related donor kidney transplantation program. Donor work-up, stress renography, transplantation, and recipient evaluation followed standard institutional protocols. The recruitment, donor assessment, transplantation, and recipient follow-up spanned 24 months from January 2023 to December 2024.

Study population

The cohort comprised 120 adult kidney donors with a baseline eGFR of 60-89 mL/min/1.73 m² and their 120 corresponding recipients. Only pairs that met the eligibility criteria and provided written informed consent were included.

Sample size calculation

Sample size estimation was performed using G*Power version 3.1 (Heinrich Heine University, Düsseldorf, Germany) [11] for multiple linear regression. A medium effect size (f² = 0.15) was assumed because no previous prospective studies evaluating stress renography-derived renal functional reserve as the primary predictor of transplant outcomes were available. Assuming up to 10 predictor variables, an alpha error of 0.05 and 80% power, the required sample size was 118 donor-recipient pairs. To compensate for possible incomplete follow-up, 120 pairs were enrolled. The final multivariable model included nine predictors. The sample size calculation was based on the primary continuous outcome of recipient eGFR at 12 months and was not specifically powered for the secondary delayed graft function or graft survival analyses.

Inclusion criteria

Eligible participants included adult kidney donors aged 18-65 years with a baseline eGFR of 60-89 mL/min/1.73 m² who were considered suitable for living kidney donation following clinical assessment, along with their corresponding recipients of living donor kidney transplantation.

Exclusion criteria

Participants were excluded if the transplantation involved a deceased donor, if they had a history of previous kidney transplantation, an active urinary tract infection, acute kidney injury, or serious systemic disease, or if relevant clinical or study data were incomplete.

Data collection

Clinical, laboratory, imaging, and operative data were recorded prospectively on standardized case report forms. Donor data included demographic characteristics, serum creatinine, eGFR, split renal function, and stress renography-derived GFR and RFR of the kidney selected for donation. The baseline and post-stimulation renographic GFR values used to calculate RFR therefore represented the function of the specific kidney that was subsequently transplanted. Recipient variables included perioperative parameters and post-transplant renal outcomes. eGFR was calculated using the Chronic Kidney Disease Epidemiology Collaboration (CKD-EPI) 2021 creatinine equation.

Stress renography protocol

All eligible donors underwent renal scintigraphy at rest and after physiological stimulation in accordance with the institutional protocol. Renal scintigraphy was performed using Tc-99m DTPA (111-185 MBq). Baseline and stimulated renographic GFR were measured separately for the kidney selected for donation using the Gates method. An oral protein load of cooked red meat (1.2 g/kg body weight) was administered as the physiological stimulus, and stimulated renal scintigraphy was performed 120 minutes after the protein load. RFR was calculated for the kidney selected for donation as the difference between its stimulated and baseline renographic GFR values. The kidney assessed by stress renography was the same kidney that was subsequently transplanted into the corresponding recipient. The renographic assessment included evaluation of renal uptake, time to peak activity, and tracer washout. RFR was categorized as normal (≥15), mildly reduced (10-14), moderately reduced (5-9), or severely reduced (<5) mL/min/1.73 m². These categories were used for exploratory stratification and should not be interpreted as universally validated clinical cutoff values.

Outcome measures

The primary outcome was recipient eGFR at 12 months after transplantation. Secondary renal function outcomes included recipient serum creatinine and eGFR during the early postoperative period and at one, three, and six months. Delayed graft function was defined as the requirement for dialysis within the first seven days post-transplantation.

Statistical analysis

Analyses were performed using IBM SPSS Statistics for Windows, Version 29 (Released 2022; IBM Corp., Armonk, New York). Continuous variables are summarized as mean ± SD or median (interquartile range), and categorical variables as frequencies and percentages. Data completeness was assessed before statistical analysis; cases with incomplete donor or recipient outcome data were excluded prior to analysis. No missing data were present in the final analytical dataset; therefore, no imputation procedures were required. Prior to inferential analyses, the assumptions underlying each statistical procedure were evaluated: normality of continuous variables was assessed using the Shapiro-Wilk test together with histogram and Q-Q plot inspection, homoscedasticity and linearity were evaluated using residual plots, and multicollinearity among predictor variables was assessed using variance inflation factors (VIF), with values below 5 considered acceptable. No clinically important multicollinearity was identified, with all VIF values below 5. Baseline eGFR and serum creatinine were both retained in the multivariable model because they represent routinely used but complementary measures of baseline renal function, and their simultaneous inclusion did not indicate problematic multicollinearity based on the prespecified VIF threshold.

The association between donor RFR and recipient serum creatinine was assessed using Pearson correlation. Multivariable linear regression was used to identify factors independently associated with recipient eGFR at 12 months; standardized β coefficients are reported to permit comparison of predictor strength, together with the model R². Model adequacy was assessed using residual diagnostics, standardized residual plots, and Cook's distance to identify influential observations. The discriminative ability of RFR, baseline eGFR, and serum creatinine for delayed graft function was compared using the area under the ROC curve (AUC) with the nonparametric DeLong test. Kaplan-Meier survival analysis with the log-rank test was used to assess graft survival, and the proportional hazards assumption was evaluated graphically before comparing survival. A two-sided p < 0.05 was considered statistically significant.

Ethical considerations

The study protocol was approved by the Institutional Ethics Committee of Fortis Hospital, Mohali, before recruitment. Written informed consent was obtained from all donors and recipients before enrollment. Participants were informed about the study procedures, potential risks and benefits, voluntary participation, confidentiality, and their right to withdraw from the study without affecting their clinical care. The study was conducted in accordance with the Declaration of Helsinki.

## Results

Study flow and participant characteristics

Of 128 donor-recipient pairs screened, 120 were enrolled after the eligibility criteria were applied. Eight pairs were excluded: three donors had an eGFR below 60 mL/min/1.73 m², two withdrew consent, and three had incomplete stress renography. Participant screening, enrollment, and follow-up are summarized in Figure 1. All participants were followed until death, graft loss, or completion of the 12-month follow-up; no participants were lost to follow-up after enrollment.

**Figure 1:**
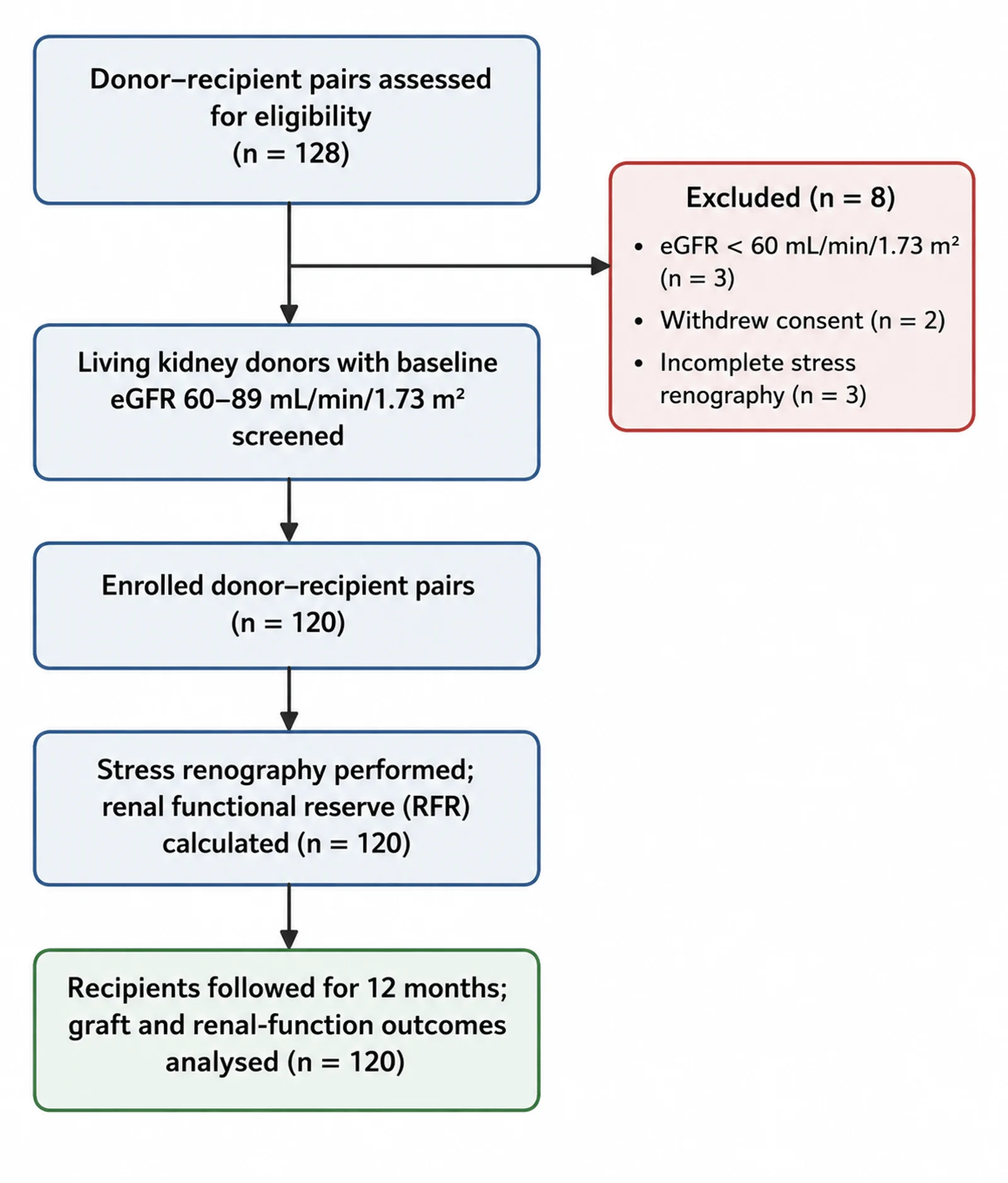
STROBE flow diagram of participant selection STROBE: Strengthening the Reporting of Observational Studies in Epidemiology; eGFR: estimated glomerular filtration rate; RFR: renal functional reserve.

Baseline characteristics of living kidney donors

The baseline characteristics of the 120 donors are presented in Table 1. The mean donor age was 44.8 ± 10.7 years, and women predominated (n = 68, 56.7%). The mean eGFR was 74.6 ± 8.1 mL/min/1.73 m², consistent with suboptimal but acceptable function for donation. Most donors were biologically related to the recipient, and the left kidney was procured in the majority of cases (Table 1).

**Table 1:** Baseline demographic and clinical characteristics of living kidney donors (n = 120) eGFR: estimated glomerular filtration rate; SD: standard deviation.

Variable	n	Mean ± SD/n (%)
Age (years)	120	44.8 ± 10.7
Sex		
Male	52	43.3%
Female	68	56.7%
Body mass index (kg/m²)	120	25.3 ± 3.2
Relationship to recipient		
Parent	28	23.3%
Sibling	46	38.3%
Spouse	22	18.3%
Other relative	24	20.0%
Systolic blood pressure (mmHg)	120	122.6 ± 9.8
Diastolic blood pressure (mmHg)	120	77.4 ± 6.5
Serum creatinine (mg/dL)	120	0.94 ± 0.13
Baseline eGFR (mL/min/1.73 m²)	120	74.6 ± 8.1
Kidney selected for donation		
Left kidney	82	68.3%
Right kidney	38	31.7%
Hypertension	8	6.7%
Diabetes mellitus	0	0.0%
Smoking history	11	9.2%

Baseline characteristics of kidney transplant recipients

Recipient characteristics are shown in Table 2. Among the 120 recipients (mean age 42.6 ± 11.3 years, n = 76, 63.3% male), chronic glomerulonephritis and diabetic nephropathy were the leading causes of ESRD. The mean pre-transplant dialysis duration was 18.4 ± 9.6 months. All transplants were ABO-compatible, with a mean HLA mismatch of 2.8 ± 1.1, and induction immunosuppression was administered in accordance with institutional policy (Table 2).

**Table 2:** Baseline demographic and transplant characteristics of kidney transplant recipients (n = 120) ESRD: end-stage renal disease; HLA: human leukocyte antigen; ATG: anti-thymocyte globulin; SD: standard deviation.

Variable	n	Mean ± SD/n (%)
Age (years)	120	42.6 ± 11.3
Sex		
Male	76	63.3%
Female	44	36.7%
Body mass index (kg/m²)	120	24.7 ± 3.5
Primary cause of ESRD		
Chronic glomerulonephritis	34	28.3%
Diabetic nephropathy	29	24.2%
Hypertensive nephrosclerosis	22	18.3%
Polycystic kidney disease	14	11.7%
Chronic interstitial nephritis	11	9.2%
Other causes	10	8.3%
Duration of dialysis (months)	120	18.4 ± 9.6
Pre-emptive transplantation	18	15.0%
HLA mismatch (number)	120	2.8 ± 1.1
ABO compatibility (compatible)	120	100.0%
Cold ischemia time (minutes)	120	68.5 ± 15.2
Warm ischemia time (minutes)	120	3.9 ± 1.2
Induction immunosuppression		
Basiliximab	74	61.7%
Anti-thymocyte globulin (ATG)	46	38.3%

Stress renography findings

Stress renography results for the 120 donors are summarized in Table 3. The mean renographic GFR increased from 74.6 ± 8.1 to 89.8 ± 10.4 mL/min/1.73 m² after stimulation, yielding a mean RFR of 15.2 ± 4.3 mL/min/1.73 m². By RFR category, 72 donors (60.0%) had RFR ≥15 mL/min/1.73 m², 34 (28.3%) had RFR of 10-14 mL/min/1.73 m², 12 (10.0%) had RFR of 5-9 mL/min/1.73 m², and 2 (1.7%) had RFR <5 mL/min/1.73 m² (Table 3).

**Table 3:** Stress renography parameters and renal functional reserve of living kidney donors (n = 120) GFR: glomerular filtration rate; T½: half-time; SD: standard deviation; RFR: renal function reserve.

Variable	n	Mean ± SD/n (%)
Baseline renographic GFR of selected kidney (mL/min/1.73 m²)	120	74.6 ± 8.1
Post-stimulation renographic GFR of selected kidney (mL/min/1.73 m²)	120	89.8 ± 10.4
Renal functional reserve of selected kidney (mL/min/1.73 m²)	120	15.2 ± 4.3
Percentage increase in GFR (%)	120	20.4 ± 6.1
Split renal function: left kidney (%)	120	50.8 ± 3.6
Split renal function: right kidney (%)	120	49.2 ± 3.6
Time to peak activity (minutes)	120	4.2 ± 0.9
Washout half-time, T½ (minutes)	120	8.6 ± 2.1
Stress renography interpretation		
RFR ≥15 mL/min/1.73 m²	72	60.0%
RFR 10–14 mL/min/1.73 m²	34	28.3%
RFR 5–9 mL/min/1.73 m²	12	10.0%
RFR <5 mL/min/1.73 m²	2	1.7%

Early post-transplant outcomes

Early graft function and clinical outcomes are shown in Table 4. Recipient serum creatinine declined from 3.12 ± 0.74 mg/dL on postoperative day 1 to 1.36 ± 0.39 mg/dL on day 7, and the day-7 eGFR was 61.8 ± 13.4 mL/min/1.73 m². Delayed graft function occurred in 11 recipients (9.2%), whereas 109 (90.8%) achieved no delayed graft function. Donor RFR was inversely related to recipient serum creatinine on postoperative day 7; higher donor RFR corresponded to lower recipient creatinine (r = −0.54, p < 0.001; Figure 2, Table 4).

**Table 4:** Early post-transplant graft function and clinical outcomes of kidney transplant recipients (n = 120) Postoperative complication categories were mutually exclusive. GFR: glomerular filtration rate; SD: standard deviation.

Variable	N	Mean ± SD/n (%)
Serum creatinine (mg/dL)		
Day 1	120	3.12 ± 0.74
Day 3	120	2.08 ± 0.61
Day 7	120	1.36 ± 0.39
Estimated GFR, day 7 (mL/min/1.73 m²)	120	61.8 ± 13.4
Urine output, day 1 (mL/day)	120	4250 ± 860
Delayed graft function	11	9.2%
No delayed graft function	109	90.8%
Biopsy-proven acute rejection	7	5.8%
Postoperative hospital stay (days)	120	8.7 ± 2.3
Postoperative complications		
None	101	84.2%
Surgical site infection	8	6.7%
Urinary leak	4	3.3%
Lymphocele	5	4.2%
Vascular complications	2	1.7%

**Figure 2:**
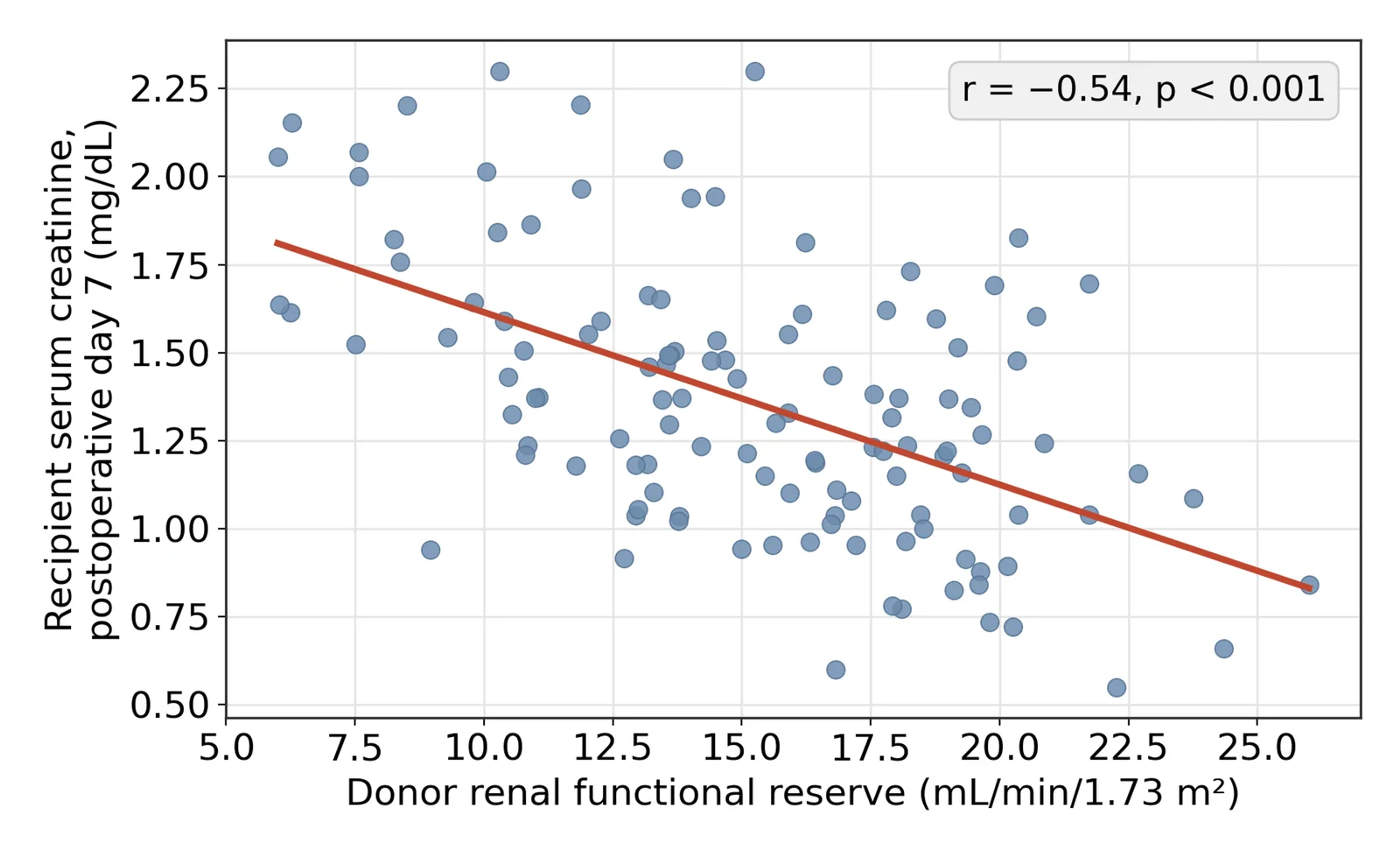
Correlation between donor renal functional reserve and recipient serum creatinine at postoperative day 7 Higher donor renal functional reserve was associated with lower recipient serum creatinine (inverse correlation).

Follow-up renal function

Twelve-month trends in recipient renal function are shown in Table 5. Serum creatinine decreased progressively, while the mean eGFR increased from 67.8 ± 12.5 mL/min/1.73 m² at one month to 74.3 ± 10.6 mL/min/1.73 m² at 12 months. Proteinuria, biopsy-proven acute rejection, and graft loss occurred in 9 (7.5%), 8 (6.7%), and 3 (2.5%) recipients, respectively. Recipient eGFR trajectories stratified by donor RFR category are shown in Figure 3.

**Table 5:** Recipient renal function and clinical outcomes during 12-month follow-up (n = 120) ᵃCumulative total, comprising the 7 early rejections reported in Table 4 plus 1 later rejection occurring after postoperative day 7. Two recipients died during follow-up; one death occurred in a recipient who experienced graft loss, while the other occurred with a functioning graft. Three graft-loss events occurred overall. eGFR: estimated glomerular filtration rate; SD: standard deviation.

Follow-up parameter	n	Mean ± SD/n (%)
Serum creatinine (mg/dL)		
1 month	120	1.24 ± 0.32
3 months	120	1.18 ± 0.28
6 months	120	1.14 ± 0.26
12 months	120	1.11 ± 0.24
eGFR (mL/min/1.73 m²)		
1 month	120	67.8 ± 12.5
3 months	120	70.6 ± 11.8
6 months	120	72.9 ± 11.2
12 months	120	74.3 ± 10.6
Proteinuria (>300 mg/day)	9	7.5%
Biopsy-proven acute rejectionᵃ	8	6.7%
Graft loss	3	2.5%
Alive at 12 months	118	98.3%
Functioning graft at 12 months	117	97.5%

**Figure 3:**
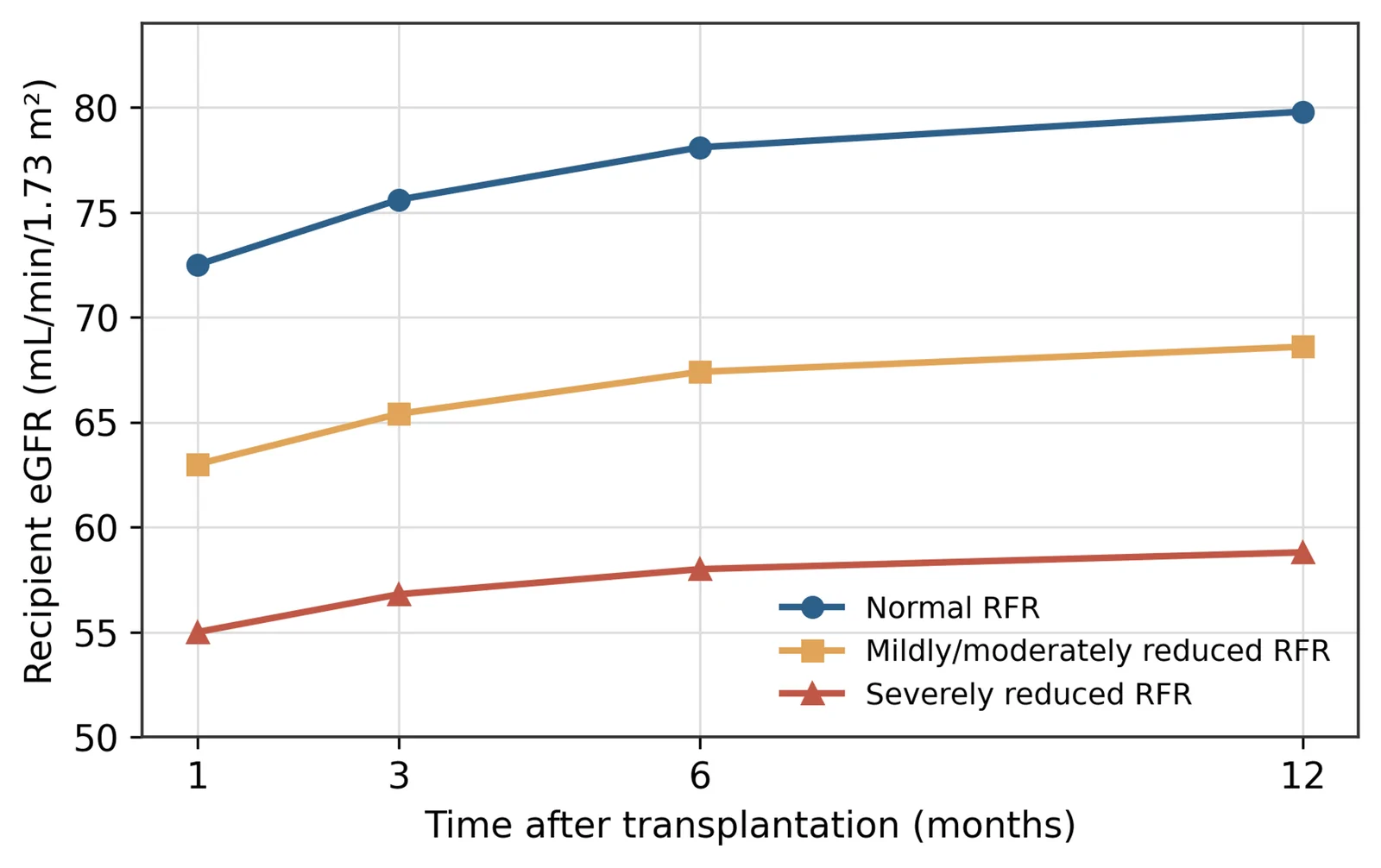
Longitudinal changes in recipient estimated glomerular filtration rate (eGFR) during 12-month follow-up by donor renal functional reserve group Recipients of kidneys from donors with higher renal functional reserve maintained higher eGFR throughout follow-up. eGFR: estimated glomerular filtration rate; RFR: renal functional reserve.

Factors associated with transplant outcomes

RFR demonstrated the largest standardized regression coefficient among the variables included in the final multivariable model and was independently associated with recipient eGFR at 12 months (standardized β = 0.487, p < 0.001; model R² = 0.45). Baseline eGFR, split renal function, donor age, HLA mismatch, cold ischemia time, donor BMI, serum creatinine, and recipient BMI were also independently associated with recipient eGFR at 12 months. Donor sex and kidney laterality were not independently associated with recipient renal outcomes and were therefore not retained in the final regression model. The small number of graft-loss events (n = 3), together with the small numbers of participants within some RFR categories, limits the precision and interpretability of the Kaplan-Meier estimates and the log-rank comparison. Therefore, the observed association between donor RFR and graft survival should be considered highly exploratory and should not be overinterpreted. RFR showed the highest AUC among the three measures (0.82 vs. 0.65 for baseline eGFR and 0.61 for serum creatinine). The difference between RFR and baseline eGFR was statistically significant by the DeLong test (p = 0.014) (Figure 6, Table 6). 

**Table 6:** Multivariable linear regression analysis of factors associated with recipient eGFR at 12 months (n = 120) β coefficients are standardized. SE: standard error; CI: confidence interval; eGFR: estimated glomerular filtration rate; BMI: body mass index; HLA: human leukocyte antigen.

Predictor variable	β coefficient	SE	95% CI	p-value
Donor age (years)	−0.152	0.041	−0.233 to −0.071	0.001
Donor BMI (kg/m²)	−0.118	0.036	−0.189 to −0.047	0.004
Baseline eGFR (mL/min/1.73 m²)	0.246	0.062	0.124 to 0.368	<0.001
Serum creatinine (mg/dL)	−0.184	0.054	−0.290 to −0.078	0.002
Split renal function of selected kidney (%)	0.134	0.043	0.050 to 0.218	0.006
Renal functional reserve	0.487	0.071	0.348 to 0.626	<0.001
HLA mismatch	−0.141	0.045	−0.229 to −0.053	0.005
Cold ischemia time (minutes)	−0.127	0.039	−0.204 to −0.050	0.004
Recipient BMI (kg/m²)	−0.096	0.034	−0.163 to −0.029	0.009

**Figure 4:**
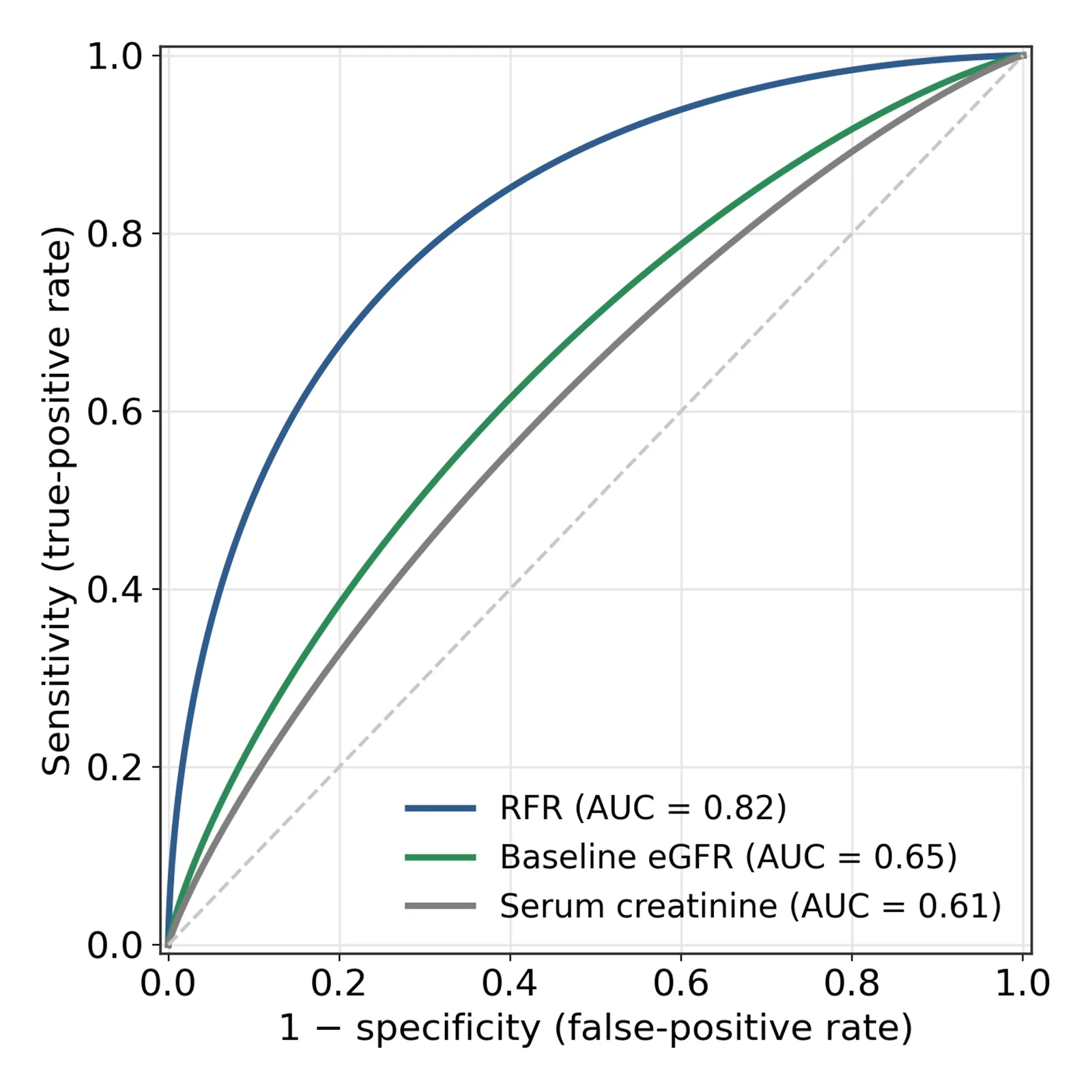
ROC curves comparing the discrimination of renal functional reserve, baseline estimated glomerular filtration rate, and serum creatinine for delayed graft function The area under the curve (AUC) was 0.82 for renal functional reserve, 0.65 for baseline estimated glomerular filtration rate, and 0.61 for serum creatinine. ROC: receiver operating characteristic; AUC: area under the curve; RFR: renal functional reserve; eGFR: estimated glomerular filtration rate.

Graft survival analysis

Kaplan-Meier graft survival stratified by donor RFR is shown in Figure 7. During the 12-month follow-up, graft loss occurred in 0 recipients in the RFR ≥15 mL/min/1.73 m² group, 1 in the 10-14 group, 1 in the 5-9 group, and 1 in the <5 mL/min/1.73 m² group. Observed graft survival was highest among recipients of kidneys from donors with RFR ≥15 mL/min/1.73 m² and decreased across the prespecified RFR categories (log-rank p = 0.002). Higher donor RFR was associated with better observed graft survival; however, these survival findings should be interpreted cautiously given the small number of graft-loss events (Figure 5).

**Figure 5:**
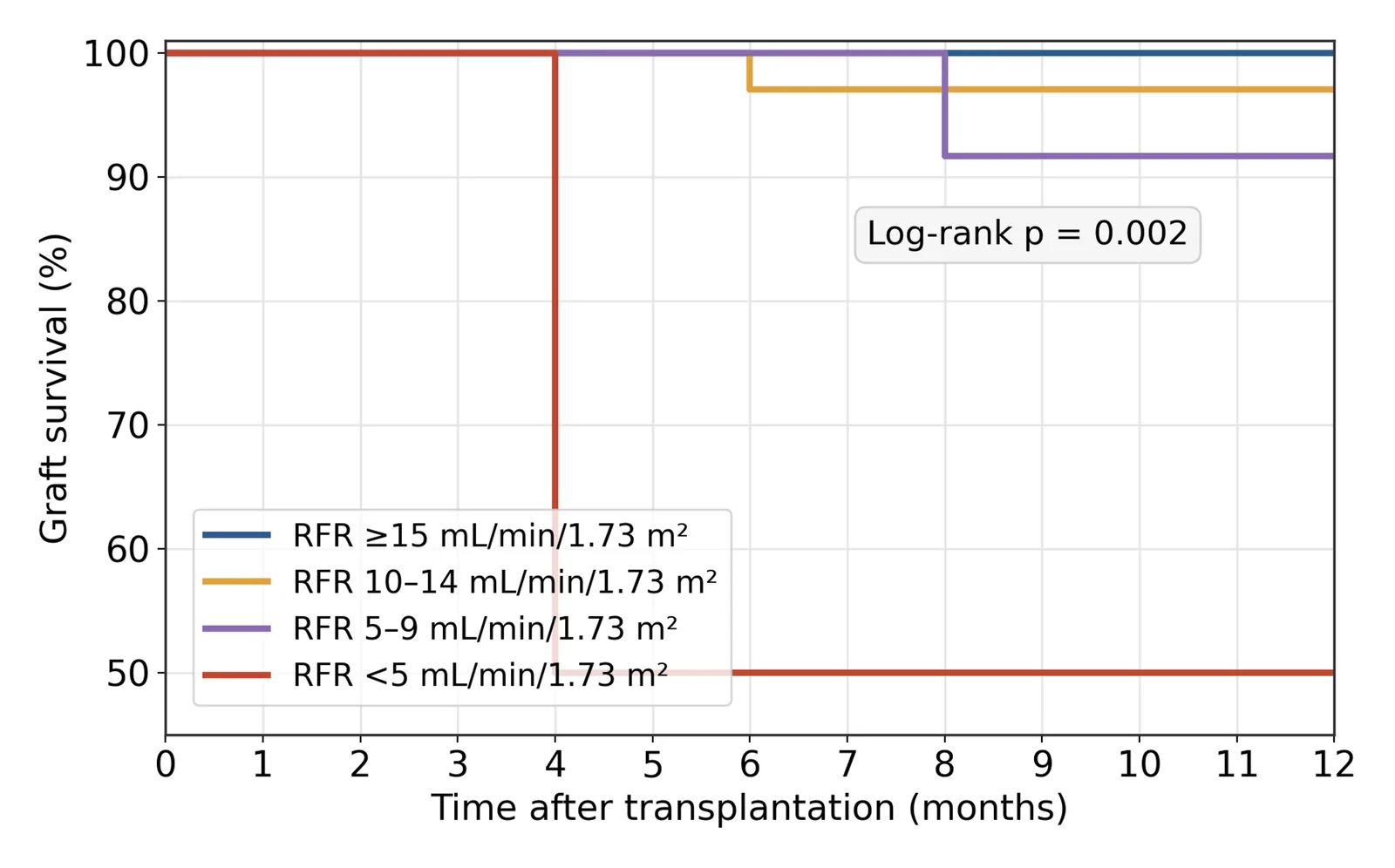
Kaplan–Meier graft survival curves by donor renal functional reserve category Graft survival was highest for recipients of kidneys from donors with RFR ≥15 mL/min/1.73 m² (log-rank p = 0.002). RFR: renal functional reserve.

## Discussion

This prospective study examined whether stress renography-derived RFR was associated with post-transplant outcomes in recipients of kidneys from living donors with suboptimal GFR. Stress renography provided functional information beyond routine assessment in the present cohort: higher donor RFR was associated with better early graft function and more favorable recipient renal recovery. Importantly, RFR remained independently associated with recipient eGFR at 12 months after adjustment for established donor and recipient factors.

Conventional donor selection depends on serum creatinine, eGFR, and split renal function, all of which characterize baseline function. Because these indices are measured under resting conditions, they provide limited information about the adaptive capacity of functioning nephrons [8]. In our cohort, stress renography demonstrated variation in RFR among donors with suboptimal baseline eGFR. The observed increase in renographic GFR after physiological stimulation is consistent with the established concept of renal functional reserve described in living-donor and renal stress-testing studies [12].

In our cohort, recipients of kidneys from donors with higher RFR had lower serum creatinine during the first postoperative week and sustained improvement in eGFR throughout the 12-month follow-up. These findings suggest that greater physiological reserve may be associated with more favorable adaptation to the increased functional demand imposed after transplantation; however, the observational design does not permit causal inference. Although earlier work has emphasized the importance of donor renal function for graft outcomes, most studies focused on parameters such as baseline eGFR, donor age, graft weight, or split renal function [13]. Because RFR was assessed for the kidney subsequently transplanted, the observed association with recipient renal function reflects the relationship between the functional reserve of the donated kidney and subsequent graft performance.

The present study extends this literature by demonstrating a potential clinical role for stress renography-derived RFR. In this cohort, RFR demonstrated a stronger independent association with recipient eGFR at 12 months than the other variables included in the multivariable model. ROC analysis also demonstrated better discrimination of delayed graft function by RFR than by baseline eGFR in our cohort. This suggests that RFR captures functional characteristics not reflected by static, biochemistry-based measures. In our cohort, recipients of kidneys from donors with preserved RFR maintained higher eGFR during follow-up, consistent with evidence that early post-transplant renal recovery trajectories are associated with subsequent renal function [14]. Kaplan-Meier analysis showed a difference in observed graft survival across RFR categories (log-rank p = 0.002). Given the small number of graft-loss events (n = 3), these survival estimates should be interpreted cautiously.

Strengths

The strengths of this study include its prospective observational design, which allowed systematic collection of donor and recipient data according to a predefined protocol. The use of a uniform institutional stress-renography protocol enabled consistent assessment of renal functional reserve across participants. Another strength was the simultaneous evaluation of donor renal functional characteristics and recipient transplant outcomes, allowing the association between donor RFR and subsequent graft function to be examined directly. The study also assessed RFR alongside conventional donor renal parameters, including baseline eGFR, serum creatinine, and split renal function, permitting comparison with routinely used measures. Furthermore, the use of multivariable linear regression, ROC analysis, and Kaplan-Meier analysis provided complementary assessments of the association between RFR and recipient renal function, its discriminatory performance for delayed graft function, and observed graft outcomes. The study evaluated clinically relevant transplant outcomes, including delayed graft function, recipient renal function, acute rejection, and graft loss, with complete 12-month follow-up and no loss to follow-up after enrollment.

Limitations

This study has several limitations. Its single-center design and use of a single institutional stress-renography protocol may limit the generalizability of the findings to other populations, transplant programs, and healthcare settings. Differences in patient characteristics, donor-selection practices, imaging protocols, physiological stimulation procedures, and methods of GFR estimation may influence RFR measurements and their interpretation. Although the study was prospective, the relatively modest sample size limited the ability to perform detailed subgroup analyses. The 12-month follow-up period also precluded assessment of long-term donor renal adaptation and graft outcomes. Furthermore, methods for assessing renal functional reserve, including the stimulus used to increase GFR and the technique used to measure GFR, are not fully standardized across institutions, which may affect the reproducibility and comparability of RFR measurements.

In the present study, renographic GFR was estimated using the Gates method rather than a reference-standard clearance technique; therefore, absolute RFR values should be interpreted in the context of the measurement method. The RFR thresholds used for exploratory stratification were not independently validated and should not be regarded as established clinical decision thresholds. Their applicability to other donor populations and institutions therefore remains uncertain. Despite adjustment for important donor and recipient variables, residual confounding inherent to the observational design cannot be completely excluded. The relatively small number of delayed graft function events (n = 11) limits the precision of the ROC estimates; therefore, the observed discriminatory performance should be considered exploratory and requires confirmation in larger cohorts. Similarly, the small number of graft-loss events (n = 3), together with the small numbers of participants within some RFR categories, limits the precision and interpretability of the Kaplan-Meier estimates and the log-rank comparison.

The observed association between donor RFR and graft survival should therefore be considered highly exploratory and should not be overinterpreted. External validation in independent cohorts was not performed, and no clinical prediction model incorporating RFR and other established predictors was developed or externally validated. Accordingly, stress renography-derived RFR should currently be considered an adjunct to conventional donor evaluation rather than a standalone criterion for donor selection or transplant prognostication. Larger multicenter studies using standardized protocols, longer follow-up, and validated RFR thresholds are required before routine clinical implementation can be recommended.

## Conclusions

Stress renography-derived renal functional reserve provided additional prognostic information beyond conventional renal-function assessment in living kidney donors with suboptimal baseline GFR. Higher RFR was independently associated with better recipient eGFR at 12 months and demonstrated better discrimination of delayed graft function than baseline eGFR. Higher RFR was also associated with better observed graft survival, although this finding should be considered exploratory because only three graft-loss events occurred. Larger multicenter studies with longer follow-up are needed to validate these findings before routine clinical implementation can be recommended.
